# Specialization Can Drive the Evolution of Modularity

**DOI:** 10.1371/journal.pcbi.1000719

**Published:** 2010-03-26

**Authors:** Carlos Espinosa-Soto, Andreas Wagner

**Affiliations:** 1 Department of Biochemistry, University of Zurich, Zurich, Switzerland; 2 The Swiss Institute of Bioinformatics, Basel, Switzerland; 3 The Santa Fe Institute, Santa Fe, New Mexico, United States of America; MRC Laboratory of Molecular Biology, United Kingdom

## Abstract

Organismal development and many cell biological processes are organized in a modular fashion, where regulatory molecules form groups with many interactions within a group and few interactions between groups. Thus, the activity of elements within a module depends little on elements outside of it. Modularity facilitates the production of heritable variation and of evolutionary innovations. There is no consensus on how modularity might evolve, especially for modules in development. We show that modularity can increase in gene regulatory networks as a byproduct of specialization in gene activity. Such specialization occurs after gene regulatory networks are selected to produce new gene activity patterns that appear in a specific body structure or under a specific environmental condition. Modules that arise after specialization in gene activity comprise genes that show concerted changes in gene activities. This and other observations suggest that modularity evolves because it decreases interference between different groups of genes. Our work can explain the appearance and maintenance of modularity through a mechanism that is not contingent on environmental change. We also show how modularity can facilitate co-option, the utilization of existing gene activity to build new gene activity patterns, a frequent feature of evolutionary innovations.

## Author Summary

Throughout life's history, organisms have produced evolutionary innovations, features that are useful when facing new ecological and environmental challenges. A property that aids in the production of such innovations is modularity. Modular systems consist of groups of molecules with many interactions within a group but fewer interactions between groups. Such modularity increases the chances of innovation, because it allows changes inside one module without perturbing others, and because it permits redeployment of modules to create new biological functions. We simulate the evolution of gene networks known to be important in development to show that modularity increases when selection favors specialization in gene activity. Specialization occurs wherever new cell types, organs, or other body structures arise. In the course of this process gene networks acquire the ability to produce new gene activity patterns specific to these structures. We also demonstrate how modularity favors the evolution of new gene activity patterns that make use of already existing modules. Because specialization in gene activity is very common in evolution, the mechanism that we put forward may be important for the origins of modularity in gene regulatory networks.

## Introduction

Most functions and structures in cells and organisms can be decomposed into smaller elements that are organized into modules [1],[2]. Such modules exist on many levels of organization, ranging from proteins and RNA to complex organs [3]. A module is a group of elements (transcription factors, signaling proteins, etc.) whose interactions occur preferentially within the group. Such an arrangement means that the activity of elements within a module depends little on elements outside of it. Thus, a module can also be viewed as a semi-autonomous entity that evolves, functions or participates in development (or other processes) relatively independently from other modules [2],[4],[5]. Modularity can enhance evolvability, an organism's capacity to generate adaptive heritable variation, for two reasons. First, the organization of biological systems into modules may permit changes inside one module without perturbing other modules. Second, modules can be combined and reused to create new biological functions [6]–[8].

Despite much recent interest in modularity [3], there is no consensus on the mechanisms that could explain its evolution [7]. Several scenarios have been proposed for the origin and maintenance of modules [9]. Two scenarios stand out, because they require conditions that organisms may encounter especially frequently. The first involves a combination of directional selection and stabilizing selection [2],[8], the second involves modularly-varying evolutionary goals [10].

Modularity might result from directional selection favoring change in one trait while stabilizing selection maintains other traits unchanged [2],[8]. Correlations between different traits can hamper both the favorable constancy of some traits and the change that in other traits would be beneficial. Under this scenario, modularity arises because the combination of directional and stabilizing selection breaks pleiotropic interactions that cause fitness trade-offs between several traits, thus allowing an escape from adaptive constraints [8],[11]. During most adaptive evolution only a few traits change while many traits are under stabilizing selection [2],[8]. Therefore, this mechanism may be a common way to evolve modularity. However, despite its eminent plausibility, population genetics models aiming to use this mechanism to produce an increase in modularity fail to do so. The reason may be their overly simple genotype-phenotype map [7].

An environment that fluctuates modularly may pose alternative evolutionary goals composed of similar sub-goals to an organism. According to previous research, such modular fluctuations in evolutionary goals can be sufficient to produce and maintain modularity [10]. In support of this scenario's importance speaks the fact that environmental fluctuation is ubiquitous. Examples include temporal variation in nutrient availability, temperature changes, changes in salinity, and many other environmental factors. Metabolic networks of bacteria living in changing environments are usually more modular than those of bacteria living in stable environments, an observation that also supports the modularly-varying goals scenario [12]. This scenario is the best current candidate for the origin of modularity in fluctuating environments. At the same time, the requirement of frequent changes in adaptive goals to maintain modularity [10] makes this scenario irrelevant where environmental demands do not fluctuate. This holds for many developmental and morphological traits. For instance, the gene network that is responsible for segment polarity in *Drosophila melanogaster* and other insects is a prominent example of a robust module in gene networks [13]–[15]. Genes in this network seem to perform similar functions in a wide range of taxa where segments are generated through otherwise disparate processes [13],[16]. It seems unlikely that such a network retains its independence from other factors because environmental demands on the fly's segments fluctuate modularly to this day. In addition, although environmental change is certainly frequent, the extent to which environments vary *modularly* is unclear. Hence, the importance of the fluctuating-environment mechanism in creating modularity is not yet proven.

Questions about modularity are questions about the structure and organization of the processes that construct phenotypic traits. Development unfolds through a sequence of gene expression states that play a major role in determining phenotypic traits [17]. In this progression some genes affect each other's activity in a network-like manner. The gene regulatory networks formed by these genes often behave modularly. For example, they maintain their intrinsic behavior even when perturbed externally, or when functioning in different contexts, such as the different parts of an organism [13], [18]–[21]. The gene activity patterns they produce are specific to particular regions of the organism or to different stages of development, and they drive specific developmental events.

In this contribution, we study the conditions under which a gene regulatory network becomes partitioned into different semi-autonomous modules. We here show that modularity can arise in gene regulatory networks as a byproduct of specialization in gene activity. Such specialization involves the evolution of new gene activity patterns that arise in a specific body part or under specific environmental conditions that organisms encounter throughout their lifetime. Namely, we show that networks that attain a gene activity pattern *I* increase their modularity when selection favors a second activity pattern *II*, provided that: i) Selection still favors *I*, so that evolved networks are able to produce both *I* and *II*, and ii) Patterns *I* and *II* share the activity state of some genes. Those genes with an activity state unique to pattern *I* or *II* have roles that are specific to their location or time of expression. Modularity arises because interactions between genes with shared and specific activities obstruct either the constancy of the former or the ability of the latter to attain different combinations of activity states. Hence, such interactions are selected against, so that the dynamics of one set of genes is affected little by the dynamics of the other set. We also show that the increase in modularity in gene networks modifies developmental constraints, thus facilitating the evolution of new additional gene activity patterns that make use of already evolved modules.

### Model

For our study we consider a network of 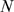 genes. Each gene's activity state is regulated by other genes in the network. The genotype of an individual is defined as the set of the interactions among its genes. We represent this set of interactions as a matrix 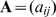. Non-zero elements in 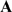 indicate activation (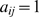) or repression (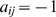) of gene 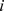 exerted by gene 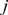. The state of the network at time 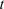 is described by a vector 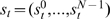. A certain gene 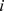 at time 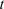 can be either active (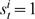) or inactive (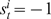). We model the change in the activity of the genes in the network according to the difference equation
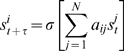
(1)where 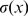 equals 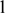 if 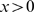, and it equals 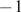 in all other cases.

Despite its simplicity, variants of this model have been successfully used to study how robustness can evolve in gene regulatory networks [22]–[24], how robustness can aid in evolutionary innovation [25],[26], and how recombination can produce negative epistasis [27]. Moreover, similar models have been successfully used to predict the dynamics of developmental processes in plants and animals [28],[29].

For our purpose, we consider that a phenotypic trait is defined by an attractor, a stable gene activity pattern resulting from the dynamics of a gene regulatory network. Attractors are often associated with developmental end-states and ‘outputs’ of developmental mechanisms [22], [30]–[32].

In order to study the evolution of modularity in gene regulatory networks, we implemented evolutionary simulations that consisted of iterative rounds of mutation and selection in populations of networks. In these simulations, we compared a set of reference gene activity patterns to actual network attractors, so that networks with attractors that were similar to the selected activity patterns had higher fitness than others (see Methods). To quantify the modularity of networks in our model, we used an algorithm [33] that identifies modules as non-overlapping densely connected groups of nodes with sparser connections between groups (see Methods). Thus, if genes in individual modules interact with many genes outside their module, the autonomy of the modules decreases, which would be reflected in a lowered modularity score.

## Results

### Specialization increases modularity

**Figure 1:**
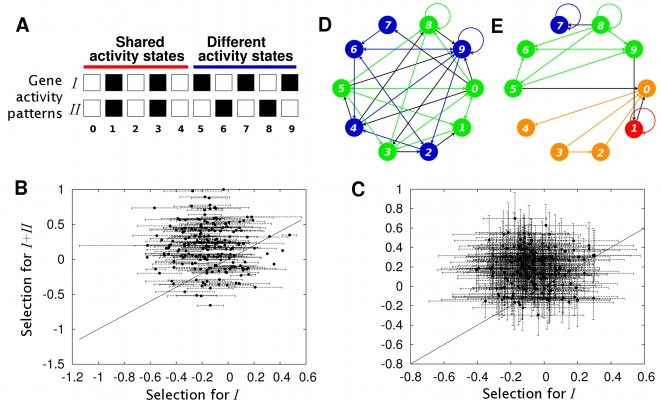
Modularity increases after selection for a new additional gene activity pattern. (A) Activity patterns *I* and *II* share the activity state of genes 0–4 and differ in that of genes 5–9. Black and white squares represent inactive and active genes, respectively. (B,C) The horizontal axes indicate mean modularity after selection for *I*. The vertical axes show modularity in networks after selection for both *I* and *II*. Specifically, (B) shows modularity of the network with highest fitness, and (C) shows mean population modularity. Points above the identity line (solid diagonal) show populations in which modularity increases after selection for the second activity pattern. The length of bars indicates one standard deviation. Plots show results for 200 evolving populations. (D,E) Nodes filled with the same color represent genes that lie in the same module. Black edges represent interactions between genes in different modules. (D) Network with the highest fitness in a population after selection for *I*. The Newman algorithm [33] partitions this network into sets in which genes 0–4 and 5–9 are intermingled. This network has a non-normalized modularity of 0.18, and a normalized modularity equal to −0.1. (E) Network with the highest fitness in a population after selection for *I* and *II*. This network is partitioned into modules in which genes with shared (genes 0–4) and different (genes 5–9) activity states in *I* and *II* lie apart. This network has a non-normalized modularity of 0.39, and a normalized modularity equal to 0.7.

To find out whether specialization can increase modularity, we studied 200 independent evolving populations of gene regulatory networks (eq. 1). Each of these populations was started with identical networks, and was subject to 500 generation cycles of mutations and selection towards attainment of a fixed-point attractor *I* (see Methods for details). The number of generations was chosen to ensure that networks that stably attain *I* can arise in the population. After gene activity pattern *I* had evolved, we allowed the population to evolve for 1500 more generations, but selecting for attainment of gene activity pattern *I* and a new pattern *II* during this time. Under this selection regime, the fittest networks were those capable of stably attaining *I* and *II* from different initial conditions that may occur in different parts of a multicellular organism. In other words, selection maintained the ability to attain *I* while at the same time favoring acquisition of *II*. Pattern *II* was chosen such that half of the network genes had identical (shared) expression states in *I* and *II*, and the other half differed in their activity state (Figure 1A). We chose such activity patterns because we hypothesized that interactions between genes with shared activity states and the rest of the genes would obstruct either i) the constant activity state of the former, or ii) the capacity of the latter to acquire different activity states independently of genes with constant activity states. If so, interactions between the different sets of genes may be selected against, thus resulting in two sets of genes with only sparse connections between them.

**Figure 2:**
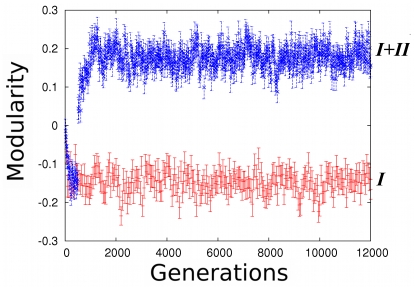
Modularity increase is not transient when selecting for a second additional gene activity pattern. Modularity in the best adapted networks reaches a plateau that is maintained for, at least, 10,000 generations when selected to attain gene activity patterns *I* and *II*. Such plateau is significantly higher than that of networks selected to attain only activity pattern *I*. The length of bars represents one standard error. The plot shows results for 100 evolving populations in each selection regime.

In most of the 200 evolving populations, modularity increased after evolving towards the attainment of both *I* and *II*. We observe this increase both in the networks with the highest fitness in the population (Figure 1B; Wilcoxon signed-rank test; 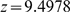; 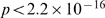), and when averaged over all networks in a population (Figure 1C; Wilcoxon signed-rank test; 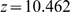; 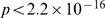). Figures 1D,E show an example of how modularity increases after selection for attainment of activity patterns *I* and *II*. Modularity does not increase when selection for *II* is absent, nor when networks evolve in the absence of selection (Figure S2). The increase in modularity is not transient because it is maintained around the same level, at least for 10,000 additional generations, when selecting for both *I* and *II* (Figure 2).

We next verified that our results were insensitive to changes in model assumptions and parameters. We first decreased the mutation rate 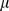, and even though the time required to evolve activity patterns *I* and *II* then increases, modularity still increases significantly (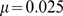; Figure S3A). Modularity increases as well when 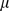 is increased (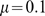; Figure S3B). We next asked whether our observations were sensitive to the assumption that individual gene activity patterns contribute to fitness additively. Changing this assumption to multiplicative fitness contributions still leads to a significant increase in modularity (Figure S4). In addition, the increase in modularity also occurs for networks containing more genes (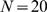; Figure S5), suggesting that such behavior does not depend on the number of genes in a network.

In a next analysis, we asked whether the increase in modularity depends on the identity of gene activity states *I* and *II*. We found that it does not, as long as some genes have the same activity state in the two patterns. For example, modularity also increases when the activity patterns differ in the activity of either three or seven genes (Figure S6A,B). Moreover, modularity increases when both the first and the second gene activity patterns are randomly chosen, except that pairs with fewer than two different activity states are discarded (Figure S6C, based on 100 populations with different pairs of activity patterns). In contrast, modularity does not increase when all genes in the activity states *I* and *II* differ in their expression (Figure S6D). This result is not due to a lack of adaptation, since networks able to attain both activity patterns arise in all evolving populations. Taken together, these observations show that modularity does not only increase for specific gene activity patterns, but that it is a generic evolutionary response. Moreover, the distinction between two sets of genes, those with identical and those with different activity in both expression patterns, is essential for the evolution of modularity. That modularity increases only in this case suggests that modules arise as a means of diminishing the effects of genes with unchanging activity on genes with changing expression in *I* and *II*, and vice versa. If so, modules should correspond to sets of genes that are required to switch their activity in a concerted manner. The following section shows that this is the case.

### Modularity partitions networks according to genes with shared and unique activity states

**Figure 3:**
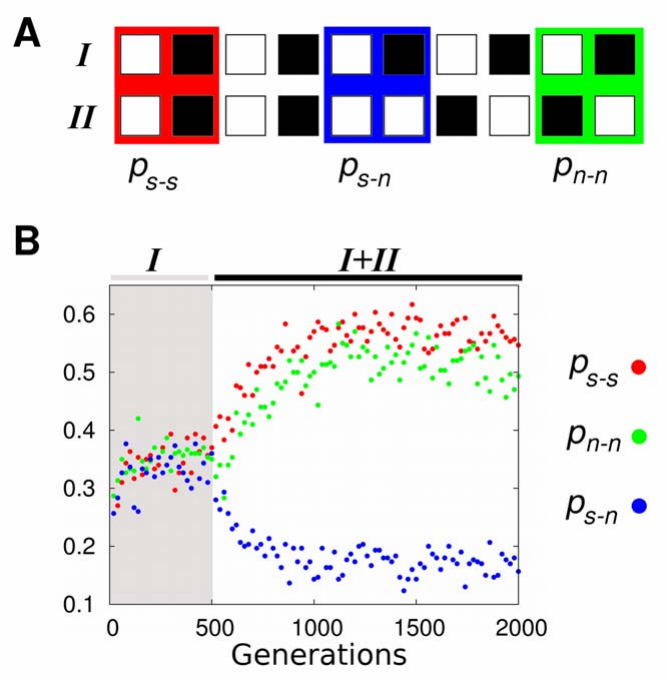
Networks become partitioned according to genes with shared and non-shared activity states. (A) 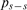 represents the frequency at which two specific genes whose activity is the same in *I* and *II* occur within the same module. 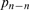 stands for the frequency at which two specific genes with non-shared activity states that change in a concerted manner occur within the same module. 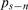 represents the frequency with which two genes, one with a shared activity state and the other with a different activity state in *I* and *II*, are in the same module. (B) As selection for activity patterns *I* and *II* starts, 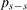 and 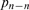 increase but 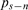 decreases. The plot shows results for 300 evolving populations.

Having established that the evolution of modularity requires genes with both shared and different activity states, we next asked whether the partitioning of modules is congruent with these two sets of genes. In other words, does one module tend to involve the genes with shared activity states, whereas another involves genes with different activity states in *I* and *II*? We evolved 300 network populations, first towards activity pattern *I* and later towards both *I* and *II*, depicted in Figure 1A. Throughout evolution, we determined for one of the best adapted networks in each population: i) the frequency 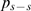 at which two genes with activity states shared in *I* and *II* occur within the same module, ii) the frequency 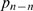 at which two genes with different activity states in *I* and *II* occur within the same module, iii) the frequency 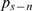 with which a specific gene with a shared activity state and a gene with a non-shared activity state are in the same module (Figure 3A). As selection for *I* and *II* occurs, 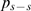 and 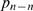 increase, while 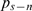 decreases (Figure 3B). This observation tells us that genes with activity states that change concertedly throughout all the selected activity patterns – be they shared or not – will tend to be included in the same module, and kept apart from other genes. This is exemplified in Figure 1D,E, which compares one of the optimal networks after selection for *I* with one of the optimal networks after selection for both *I* and *II*. The latter is partitioned into modules in which genes with shared and distinct activity states in *I* and *II* lie apart. Thus, the structure of modules reflects the manner in which selection has molded the traits, as has been previously suggested [2].

### Modularity increases further after selection of a third activity pattern

**Figure 4:**
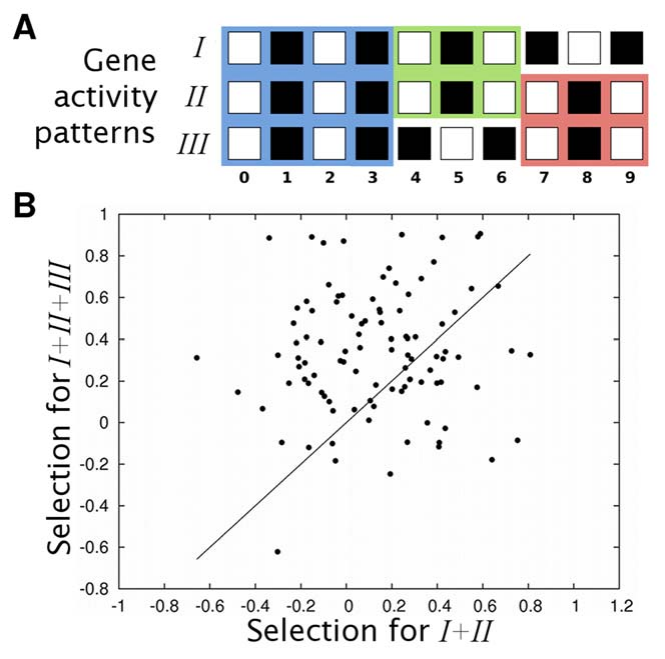
Modularity increases further after selection for a third activity pattern *III*. (A) Gene activity patterns *I*, *II* and *III*. White squares represent active genes and black squares represent inactive genes. Background color distinguishes genes that change their activity state in a concerted manner across all selected patterns. Notice that, in this case, the inclusion of additional activity patterns results in more and smaller groups of genes whose activity changes concertedly. (B) The horizontal axis indicates modularity of the best adapted networks after selection for *I* and *II*. The vertical axis shows modularity of the best adapted networks after an additional 3000 generations of selection for *I*, *II* and *III*. Wilcoxon signed-rank test; 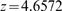; 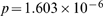. The plot shows results for 100 evolving populations.

We also tested whether modularity arises only where selection favors the attainment of two gene activity patterns, or whether it increases further with even more gene activity patterns. To this end, we analyzed 100 evolving populations in which selection first favored a gene activity pattern *I* (500 generations), then an additional pattern *II* (*I+II*, next 1,500 generations), and then a third pattern *III* (*I+II+III*, last 3,000 generations). The patterns share the activity of some genes and differ in others. As selection for the third pattern begins, more and smaller groups of genes arise whose activity changes in a concerted manner (Figure 4A). Interactions between different such groups would obstruct evolutionary adaptation. Such interactions should thus be selected against, resulting in a further increase in modularity. Our observations confirm this hypothesis. After selection for patterns *I* and *II*, we observed a significant first increase in modularity (Wilcoxon signed-rank test; 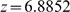; 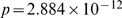). Modularity increased further after selection for pattern *III* (Figure 4B; Wilcoxon signed-rank test; 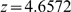; 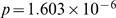).

In addition, we observed an increased number of modules in networks with high fitness after selection for patterns *I* and *II*. Moreover, this number increases further after selection for patterns *I*, *II* and *III* (Figure S8B). This result suggests that the increase in modularity after selection for the three patterns occurs because of the appearance of new modules, and is not a mere consequence of the consolidation and refinement of previously evolved modules. We also analyzed how the probability of two genes being part of the same module changes across evolution. We found that the frequency of two genes occurring in the same module in the fittest networks of each evolving population changes according to whether those genes change their activity concertedly across the selected patterns (Figure S8C). For example, as we depict in Figures 4 and S8A, the activity of genes 5 and 6 changes concertedly across all activity patterns: if in one pattern gene 5 is active, then gene 6 is inactive in that same pattern, and vice versa. The frequency with which those genes lie in the same module increases across evolution. In contrast, the activity of genes 0 and 6 changes concertedly when selecting for patterns *I* and *II*, but not when also selecting for activity pattern *III*. Thus, the probability of those genes occurring in the same module increases prior to selection for pattern *III*. After selection for pattern *III* starts, the probability that genes 0 and 6 lie in the same module decreases abruptly (Figure S8C). These results show that the modules that arise after selection for the third pattern also tend to coincide with sets of genes whose activity states change concertedly throughout the selected patterns. Computational cost did not allow exploration of further increases in modularity via selection of additional gene activity patterns. However, our observations already suggest that modularity will increase as long as there is an increase in the number of gene groups for which concerted activity changes are favored.

### Modularity facilitates co-option

**Figure 5:**
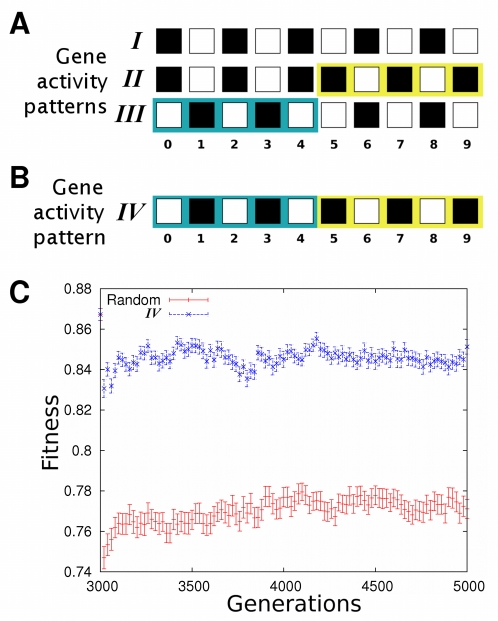
Maximal fitness increases faster when co-option of existing gene activity states is possible. (A,B) Black and white squares represent inactive and active genes, respectively. (A) Networks first attained activity patterns *I*, *II* and *III* after 3,000 generations of evolution. The selection regime promotes the evolution of modules containing genes 0–4 on one hand, and genes 5–9 on the other. (B) After 3,000 generations, selection favored gene activity pattern *IV*, which is a combination of activity patterns matching those of previously evolved modules, as indicated by the background colors. (C) Networks selected to attain a fourth activity pattern increase their fitness much faster if this pattern is *IV*, than if it is a randomly chosen activity pattern. The length of bars indicates one standard error.

A question recurring in the literature is how modularity may increase evolvability by facilitating co-option, the combination of previously evolved modules to perform new functions [19]–[21], [34]–[36]. We addressed how the previous evolution of modules in gene regulatory networks biases future evolutionary potential by asking whether gene networks acquire new gene activity patterns faster if these patterns use gene activity states associated with previously evolved modules. Specifically, we selected networks for their ability to stably attain three gene activity patterns *I*, *II* and *III* (Figure 5A). We chose the specific combination of patterns in Figure 5A because: i) it promotes the evolution of a module including genes 0–4 and another module including genes 5–9, as shown above, and, ii) it allows the inclusion of an additional activity pattern (*IV*) that is composed entirely of activity states associated with previously evolved modules (Figure 5A,B). After 3,000 generations, we subjected networks in 100 evolving populations to selection favoring such an additional gene activity pattern *IV* (Figure 5B). Importantly, this pattern shares the activity states of genes 0–4 with *III*, and the activity state of genes 5–9 with *II*. Thus, gene activity pattern *IV* may evolve by combining previously evolved modules in a new manner. In addition, we repeated this approach in 100 “control” populations where the fourth favored gene activity pattern was randomly chosen with equal probability for genes being active and inactive. Notice that we do not expect that selection for activity pattern *IV* increases modularity, because the inclusion of this pattern does not cause an increase in the number of gene groups with concerted activity changes. Rather, we hypothesize that modularity facilitates the evolutionary acquisition of such an activity pattern, as compared to other activity patterns.

We found that networks with high fitness arise much more rapidly when *IV* is the new gene activity pattern. This indicates that pattern *IV* is much easier to attain than random gene activity patterns in populations of networks that have previously been selected for their ability to attain *I*, *II* and *III* (Figure 5C). The same trend occurs when not just the networks with highest fitness are considered, but also when we analyze mean population fitness (Figure S7). We note that in our analysis selection favors the attainment of *IV* to the same extent as the attainment of any one random gene activity pattern in the control populations. This means that our observations are not simply caused by a greater increase in fitness conveyed by *IV*. The fitness increase rather depends on how easily the new gene activity patterns can be constructed: it is easier to evolve gene activity patterns that combine activity states of previously evolved modules.

## Discussion

In sum, we showed here that modularity arises in gene networks when they acquire the ability to attain new activity patterns that share the activity state of some genes with old patterns. Our observations indicate that selection to attain the new activity patterns can cause modularity to arise in gene regulatory networks when pleiotropic effects obstruct adaptation [2],[8],[11]. Such pleiotropic effects are caused by interactions between (i) genes whose activity is shared between different patterns, and (ii) genes whose activity is specific to one pattern: If changes in the latter affect the former, evolutionary acquisition of the new pattern is hindered. Thus, the scenario we propose favors networks with few interactions between genes with an unchanging activity state and genes that adopt new regulatory functions. In this way, genes that have correlated activity states come to lie in the same network module (Figures 3 and S8C). Our results suggest that modularity increases as long as selection favors new activity patterns involving more and smaller groups of genes whose activity changes in a concerted manner (Figures 4 and S8).

Empirical falsification (or validation) of the mechanism that we propose ideally requires comparative analyses of the structure of gene regulatory networks in several related species. Such information might not be available soon. However, existing information from various sources suggests that the mechanism we propose could be important. Specifically, the evolutionary acquisition of new gene activity states by regulatory networks is ubiquitous in evolution, and nowhere more than in the evolution of development. It occurs wherever new cell types, organs, or body structures, arise from previously undifferentiated ones. Many examples in the literature suggest that some genes exhibit specialized activity in different parts of an organism, whereas others present shared activity patterns. Indeed, gene functions may be inferred via correlated gene expression patterns in conventional or high-throughput expression analyses [37]–[39]. For example, the activity of the same genes patterns both vegetative and floral meristems in the plant *Arabidopsis thaliana*. Floral identity genes are active exclusively in floral meristems, so that the floral structure is determined by both the floral identity genes and the shared patterning genes [40]–[42]. In the sea urchin *Strongylocentrus purpuratus*, some differentiation genes are active in the micromer lineage that produces the euechinoid exclusive embryonic skeleton and also in the independently derived juvenile skeletogenic centers that produce the adult skeleton [43]. Some other genes of the gene network that specifies the skeletogenic micromere lineage are active in those cells but not in the juvenile skeletogenic centers. Examples include genes involved in induction of neighbouring cells or in triggering the initial stages of micromere specification [43],[44]. Another example involves the cellular level. Mammalian brown fat cells share some traits and gene activity patterns with white fat cells, and others with muscle cells [45]. More generally, evolutionarily derived cell types usually perform just a fraction of the functions that ancestral cell types performed [46], a trend that will lead to similar activity states for some genes and different states for others in sister cell types.

In a similar vein, evolutionary specialization of initially homogeneous metameric units is likely to occur mainly by modifications (such as changes in the transcriptional circuitry) that result in metamers with different activity states of some genes but not of others; otherwise, differentiated metameric units would be hardly recognizable as such. For example, in *D. melanogaster*, limbs are positioned and patterned by mechanisms that are reiterated along the body, however limb identity relies on segment-specific mechanisms [47]. Moreover, in heteronomous arthropods, in which the morphology of segments along an individual is very distinct, processes underlying segmentation and limb differentiation interact less than in homonomous arthropods, in which the segments along a body are very similar [47]. Segment formation is performed throughout the organism (shared), and, in heteronomous taxa, limb identity determination is specialized according to the place where a limb develops. Thus, when there is specialization in limb identity, the two processes are more independent, in contrast to taxa that lack this specialization.

Co-option, the recruitment of previously evolved modules to perform new functions, is a common feature of evolutionary innovations [20],[21],[34],[36],[43]. A case in point regards the gene network regulating pharyngeal dentition in fish, which is co-opted to also generate oral dentition [36]. Another example is the gene network that patterns the insect wing blade. It is co-opted to determine the localization of eyespots in butterfly wings [34]. Our work shows that a modular network may readily generate new gene activity patterns that make use of gene activity states of previously evolved modules. The existence of such structured, or “facilitated” variation has been known for a long time [48]–[51]. Our work provides a candidate mechanism to create such variation, namely via network modularity that results from specialization in gene activity. Our observations could thus help explain the repetitive co-option of several modules, such as that responsible for proximal-distal polarity in lateral appendages and body outgrowths [20],[21], or the *achaete* and *scute* module that operates in a wide range of developmental processes in animals [19].

An alternative hypothesis for the evolution of modularity is the ‘modularly-varying goals’ scenario [10]. This scenario requires that populations are exposed to evolutionary goals that fluctuate over time, so that modularity can arise and be maintained. In contrast, our scenario requires specialization of gene activity, that is, new gene activity patterns must be attained while old activity patterns are preserved. Relatedly, the modularly-varying goals scenario requires genetic changes for evolutionary adaptation after an evolutionary goal changes. In contrast, our mechanism requires one genotype to produce different activity patterns under different conditions, conditions that may occur in different parts of a multicellular organism. In other words, in our scenario, modularity arises to avoid obstruction to attain different selected patterns within the same genotype. Our scenario may thus be more appropriate for traits where environmental demands are not constantly fluctuating, such as in the development of many morphological traits in plants and animals.

Thus far, we motivated our approach with the development of multicellular organisms. However, the approach could also explain modularity in unicellular organisms. For example, the metabolic networks of bacteria living in changing environments tend to be more modular than those of bacteria living in stable environments [12]. Similar patterns may exist for gene regulatory networks. If so, the modularly varying goals scenario is not their only possible explanation. Unicellular organisms respond to changing environments by tuning their gene activity pattern. In other words, they usually have adaptively plastic phenotypes. For example, different sets of genes are activated or repressed when yeast cells are exposed to different environments [52]–[54]. Evolving the ability to switch gene expression according to the environment requires producing several alternative activity patterns, as we propose here. Importantly, some yeast genes change their expression concertedly in several environments, whereas others have responses that are specific to any one environment [52]–[54]. This observation suggests that the activity of some genes is shared across alternative activity patterns while the activity of other genes is particular to certain environments, as our model demands. In sum, because organisms in changing environments are required to produce different gene activity patterns according to the environment, our scenario can explain the evolution of modularity both in fluctuating and non-fluctuating environments.

A question that remains unanswered is whether our model applies to genotype-phenotype maps different from those of gene regulatory networks. A prominent example is metabolic networks, whose phenotypes are patterns of metabolic fluxes through network reactions. Our framework may apply to some instances of modularity in metabolic systems, as the following example illustrates. The main requirement of our model is an increase in the number of functions that a network must perform (i.e. in the number of selected gene activity patterns). The appearance of new functions in a metabolic network usually involves the production of new metabolites. Hintze and Adami [55] performed evolutionary simulations of an artificial metabolism in which the fittest metabolic networks were able to produce an increasingly diverse spectrum of metabolites. This selection regime resulted in increased modularity of metabolic networks, an observation consistent with the mechanism that we propose for gene regulatory networks.

Our work aimed at conceptual clarity by using only few essential assumptions in explaining the evolution of modularity. We therefore neglected many processes that doubtlessly play a major role in the evolution of regulatory gene networks. For example, we did not consider mutations changing the number of genes in a network, even though processes such as gene loss or duplication may be frequently involved in the appearance of new gene activity patterns. Similarly, the appearance of new body structures or cell types requires interactions among cells, tissues and organs. Such interactions ensure the proper placement of cells with the combination of general and specialized gene activity that is characteristic of specialization. The incorporation of these and other processes in future work will deepen our understanding of the evolution of modularity, and thus of evolvability.

## Methods

### Modularity

We here identify modularity using one [33],[56] of several algorithms aimed at identifying structural modules, densely connected groups of nodes with sparser connections between groups. The measure of modularity in this algorithm is a score 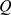 that compares the abundance of intra-module connections between a given network to that of random networks with the same degree distribution [57]. 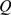 is defined as:
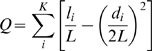
(2)where 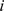 denotes one of the 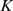 prospective modules in a network, 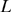 stands for the total number of edges in the network, 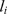 represents the number of edges within module 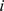, and 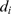 is the sum of the number of connections that each node in module 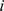 has [33], [56]–[58]. The algorithm we use [33] identifies a partitioning of networks into modules that maximizes 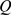. We use this algorithm because of its computational efficiency and accuracy [33],[56]. We also explored different algorithms [57],[58] and found that our results hold regardless of these choices.

Typical 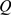 values of partitions that maximize intra-module connections in random networks vary depending on the number of nodes, edges and connectivity distribution [59]. For example, the maximum 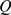 value of a network varies as a function of the total number of edges in it [60]. Hence, a fair comparison of modularity in different networks requires first addressing how atypical 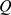 is in the best partition of each network when compared with random networks with the same attributes. Following [10] we use for normalization the equation:
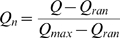
(3)where 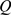 is the modularity returned by the Newman algorithm [33],[56] for a certain network, 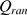 stands for the average 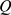 value of 1,000 random networks with the same number of genes and edges and the same degree distribution as the original network. 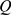 values for these random networks are also calculated using the Newman algorithm. 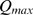 is the maximal 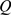 value in these 1,000 random networks. The normalized modularity 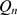 tells us how modular a network is in comparison to random networks with the same attributes. Non-normalized and normalized 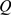 values render equivalent results in our analysis (Figure S1). Therefore, we restrict ourselves to report results for normalized modularity.

### Fitness

The fitness function we use compares a set of reference gene activity patterns to actual network attractors. Our fitness measure also incorporates the likelihood that an attractor is attained in the face of perturbations. In doing so, it takes into account not only the identity of an attractor but also its robustness, an important feature for the stability and reproducibility of developmental processes [13], [18], [61]–[63].

For each gene activity pattern 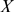 that contributes to fitness and for each network in our analysis, evaluation of fitness involved the following steps: i) The initial state of the gene network at time 0 was chosen to be a perturbation of the target pattern 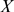, drawn from a probability distribution where the initial state of each gene differs from that of 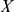 with probability 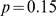. ii) We carried out network dynamics (eq. 1) until some new attractor 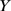 was reached; iii) We recorded the Hamming distance (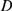) separating 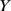 from 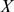, and calculated the contribution to fitness of this developmental trajectory as 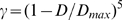; modifications of 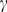 by varying the exponent produce equivalent results. iv) We repeated steps i)–iii) 500 times to determine 500 values 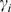 (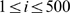). Notice that several of such 500 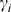 values would correspond to the same initial condition, and that the distribution of possible initial conditions is biased towards gene activity patterns similar to the reference pattern 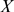. This reflects our assumption, for the sake of simplicity, that selection favors similar initial conditions leading to the same selected activity pattern. We also assumed that gene activity patterns that are similar to the reference pattern are more likely to be required as initial conditions. Relaxation of such assumptions by variation in 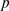 did not modify our results.

We then calculated the network's fitness as
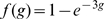
(4)where 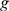 is the arithmetic mean of all 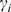. Wherever fitness needed to be evaluated for multiple gene activity patterns, we calculated the arithmetic mean of 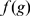 over these multiple patterns. Notice that selection is pushing the acquisition of different gene activity patterns that would appear under different conditions (such as different parts of the organism). Hence, the optimal networks will be those with dynamics that lead to different attractors matching the reference activity patterns, and not those with a single attractor that is a combination of the reference patterns.

Had we used multiplicative contributions to fitness then the benefits that result from attaining a gene activity pattern would have depended on the acquisition of all other activity patterns. Because our simulations start with selection for a single activity pattern, it was preferable to assume otherwise. Using additive contributions to fitness guarantees that networks that are not able to attain the new gene activity pattern still have a chance to contribute to the next generation. However, usage of multiplicative fitness contributions does not affect our results qualitatively.

### Evolutionary simulations

For each simulation of gene network evolution, we first built a 10 node network and added 20 interactions at random to its interaction matrix 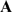. These interactions were activating or repressing, with equal probability. To construct the initial population we exposed 100 copies of this initial network to random mutation. Mutations occurred independently among different genes. A mutation of a gene either added a positive or negative interaction affecting the gene's activity, or eliminated one of the interactions that regulated the gene. Such mutations can be interpreted as changes in the regulatory regions of a gene, adding or eliminating cis-regulatory elements. Most of our results are based on a probability of a mutation occurring in a gene (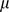) of 0.05. This value of 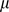 allowed adaptation within a tractable number of generations. Variation in 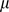 did not affect our results, but only affected the time required for adaptation. For a gene 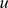 undergoing mutation, we defined the probability of losing an interaction as
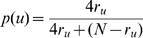
(5)and the probability of acquiring a new interaction as 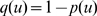. Here 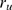 represents the number of regulators of the gene 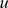, and 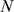 equals the number of genes in the network, and hence, the maximum number of regulators of any gene. This procedure results in networks that evolve towards low connectivities of 2–3 regulators per gene. Such low connectivity is often observed in transcriptional regulation networks of plants, animals, fungi and bacteria [64]. Loss of interactions may also help explain the observation that loss of gene expression is more common than acquiring new expression patterns in the evolution of gene families [65].

In our evolutionary simulations, we kept populations of constant size (100 individuals) and imposed iterative rounds of mutations and selection to those populations. Every new generation, we sampled networks from the ones in the previous generation with probability proportional to the networks' fitness. Specifically, we defined the probability of copying network 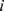 from generation 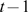 into a network 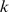 in the new generation (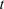) as 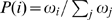, where 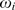 stands for the fitness of network 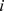. Each of the new networks underwent mutation with a probability of 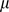 per gene. Finally, we evaluated the fitness of each new network. We iterated these steps through the end of the simulation.

All simulation code (written in C++) took advantage of the LEDA library of C++ data types [66].

## Supporting Information

Figure S1 Non-normalized modularity increases after selection for a new additional gene activity pattern. The horizontal axis indicates mean non-normalized modularity after 500 generations of selection for gene activity pattern *I*. The vertical axis shows non-normalized modularity in networks after an additional 1500 generations of selection for both gene activity patterns *I* and *II*. Specifically, (A) shows modularity of the networks with highest fitness (Wilcoxon signed-rank test; *z = 8.8597*; *p<2.2×10^−16^*), and (B) shows mean population modularity (Wilcoxon signed-rank test; *z = 10.073*; *p<2.2×10^−16^*). Points above the identity line (solid diagonal) show populations in which modularity increases after selection for the second gene expression pattern. The length of bars represents one standard deviation. The plots show results for 200 evolving populations.
(2.95 MB TIF)

Figure S2 Modularity does not increase when selection does not favor specialization. The length of bars represents one standard deviation. The plots show results for 100 evolving populations. (A) Modularity does not increase under selection for a single gene activity pattern (*I* in Figure 1A in the main text). Wilcoxon signed-rank test; *z = −0.02063*; *p = 0.50823*. The horizontal axis shows modularity in the best adapted networks after 500 generations. The vertical axis shows modularity in the best adapted networks after 2000 generations. (B) Networks evolving in the absence of selection do not increase their modularity (Wilcoxon signed-rank test; *z = −0.2882*; *p = 0.61364*). The horizontal axis indicates mean modularity after 500 generations. The vertical axis shows modularity in networks after an additional 1500 generations.
(1.00 MB TIF)

Figure S3 Modularity increases under evolution with different mutation rates. The horizontal axes indicate modularity in the best adapted networks after 1000 generations of selection for gene activity pattern *I*. The length of bars represents one standard deviation. The plots show results for 100 evolving populations. (A) Modularity increases using a mutation rate that equals half of the value used in other simulations (*μ = 0.025*; Wilcoxon signed-rank test; *z = 6.8835*; *p = 2.9194×10^−12^*). This increase occurs but requires longer time scales to achieve adaptation. The vertical axis shows modularity in the best adapted networks after an additional 3000 generations of selection for both gene activity patterns *I* and *II*. (B) Modularity increases using a mutation rate that doubles the value used in other simulations (*μ = 0.1*; Wilcoxon signed-rank test; *z = 7.4921*; *p = 3.3862×10^−14^*). The vertical axis shows modularity in the best adapted networks after an additional 1500 generations of selection for both gene activity patterns *I* and *II*.
(1.48 MB TIF)

Figure S4 Modularity increases when fitness components related to each activity pattern combine multiplicatively instead of additively. Wilcoxon signed-rank test; *z = 6.9385*; *p = 1.9809×10^−12^*. The horizontal axis indicates modularity in the best adapted networks after 500 generations of selection for gene activity pattern *I*. The vertical axis shows modularity in the best adapted networks after an additional 1500 generations of selection for both gene activity patterns *I* and *II*. The length of bars represents one standard deviation. The plot shows results for 100 evolving populations.
(0.83 MB TIF)

Figure S5 Modularity increases when evolving networks composed of twice as many genes as in other simulations. *N = 20*; Wilcoxon signed-rank test; *z = 5.1987*; *p = 1.0032×10^−7^*. (A) Activity patterns *I* and *II* share the activity state of genes 0–9, but show different activity patterns for genes 10–19. White squares represent active genes and black squares represent inactive genes. (B) The horizontal axis indicates modularity in the best adapted networks after 800 generations of selection for gene activity pattern *I*. The vertical axis shows modularity in the best adapted networks after an additional 2700 generations of selection for activity patterns *I* and *II*. We adjusted the mutation rate *μ* so that the expected number of individuals without any mutation is approximately the same as in all other simulations. Because of computational cost, we here followed 250 developmental trajectories for each network to evaluate the contribution to fitness associated to a certain gene activity pattern, instead of 500 as in our other analyses. The length of bars represents one standard deviation. The plot shows results for 100 evolving populations.
(1.70 MB TIF)

Figure S6 The increase in modularity does not depend on the identity of the selected activity patterns. The horizontal axes indicate modularity after 500 generations of selection for a single gene activity pattern. The vertical axes show modularity after an additional 1500 generations of selection for two gene activity patterns. The length of bars represents one standard deviation. The plots show results for 100 evolving populations. (A) Modularity increases in the best adapted networks when the two selected gene activity patterns differ in the activity state of 3 genes (Wilcoxon signed-rank test; *z = 6.7185*; *p = 9.1811×10^−12^*). (B) The same occurs when the two selected gene activity patterns differ in the activity state of 7 genes (Wilcoxon signed-rank test; *z = 5.6045*; *p = 1.0445×10^−8^*). (C) Modularity increases after selection for two gene activity patterns picked at random (Wilcoxon signed-rank test; *z = 5.9449*; *p = 1.3834×10^−9^*). We discarded pairs of gene activity patterns with less than two different activity states. The probability of picking a pair with *k* activity differences in a 10-gene network is *p(k) = C^10^_k_0.5^10^*, where *C^N^_k_* is the binomial coefficient. However, after discarding activity patterns with less than two different activity states, *p(k) = [C^10^_k_0.5^10^][1−(C^10^_0_+C^10^_1_)0.5^10^]^−1^*. (D) Modularity does not increase when gene activity patterns differ in the activity state of all genes (Wilcoxon signed-rank test; *z = 1.0281*; *p = 0.15196*). This result is not due to a lack of adaptation, since networks that can attain both activity patterns in a stable manner arise in all evolving populations.
(3.14 MB TIF)

Figure S7 Mean fitness increases faster when co-option of existing gene activity states is possible. Mean population fitness increases faster when selecting for a new gene activity pattern (*IV* in Figure 5 in the main text) that co-opts activity states matching those of previously evolved modules than when such activity pattern is picked at random. This shows that the increase in fitness when selecting for pattern *IV* permeates the whole population, and affects not only the best adapted networks. The length of bars represents one standard error.
(1.02 MB TIF)

Figure S8 New modules arise after selection for a third additional pattern. (A) Gene activity patterns *I*, *II* and *III*, as in Figure 4A. (B) The number of modules in the networks with the highest fitness in each population, averaged across populations, increases after selection for the new additional patterns. The length of bars represents one standard error. (C) *p_x,y_* stands for the frequency with which genes *x* and *y* occur in the same module in the networks with the highest fitness of each evolving population. When selection for a new activity pattern causes the activity of two genes to cease changing concertedly across the selected patterns, the probability of such genes lying in the same module decreases rapidly. This is the case of genes 0 and 8 after selection for activity pattern *II* starts, and also of genes 0 and 6 after selection for pattern *III* begins (red arrow). The plots show results for 100 evolving populations.
(4.06 MB TIF)
