# Supplementary figures and images for: Specialization Can Drive the Evolution of Modularity
Source: PLoS Comput Biol. 2010 Mar 26;6(3):e1000719. doi: 10.1371/journal.pcbi.1000719 (PMC2847948; doi:10.1371/journal.pcbi.1000719)

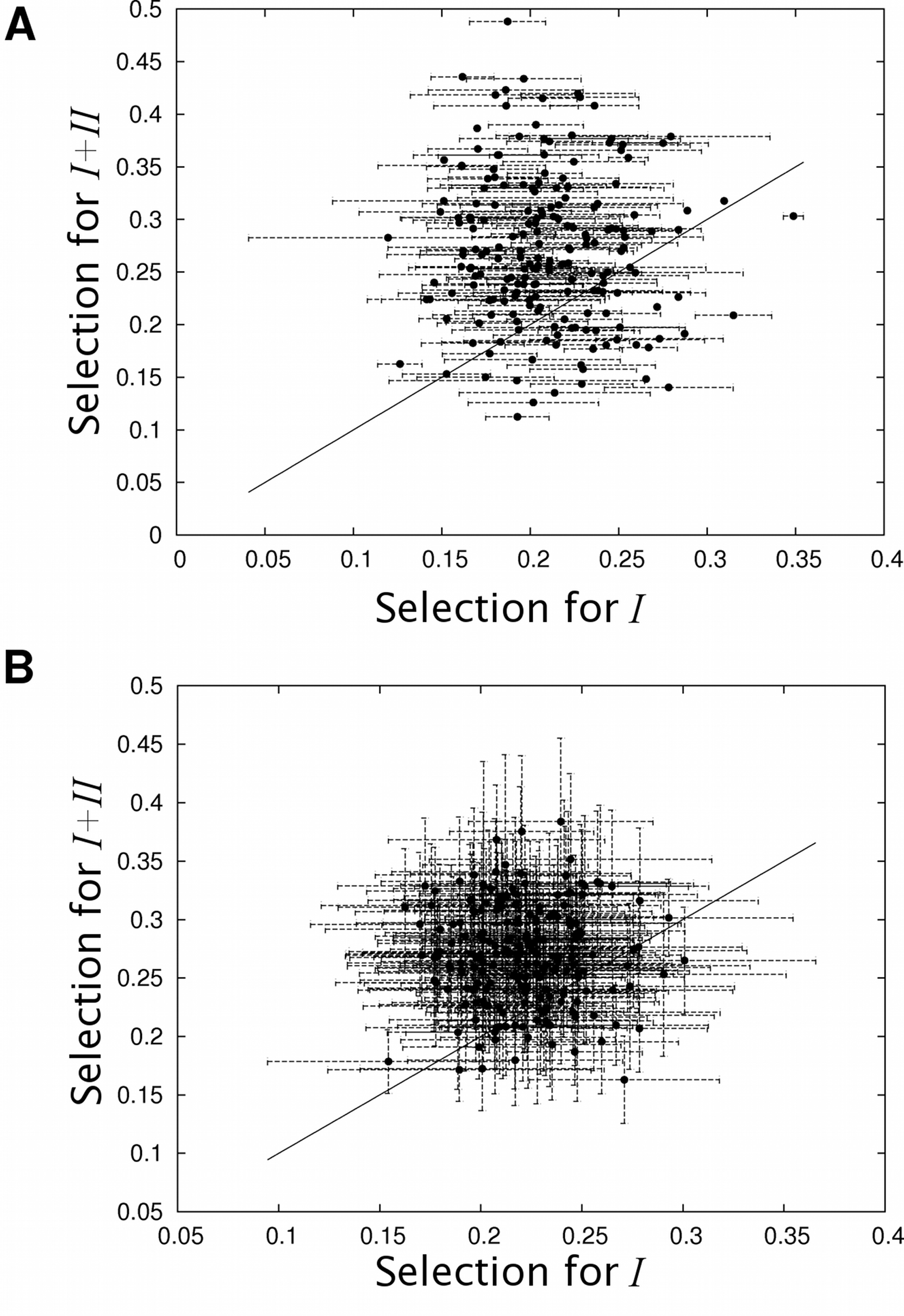

Supplement: Figure S1 — Non-normalized modularity increases after selection for a new additional gene activity pattern. The horizontal axis indicates mean non-normalized modularity after 500 generations of selection for gene activity pattern I. The vertical axis shows non-normalized modularity in networks after an additional 1500 generations of selection for both gene activity patterns I and II. Specifically, (A) shows modularity of the networks with highest fitness (Wilcoxon signed-rank test; z = 8.8597; p<2.2×10^−16^), and (B) shows mean population modularity (Wilcoxon signed-rank test; z = 10.073; p<2.2×10^−16^). Points above the identity line (solid diagonal) show populations in which modularity increases after selection for the second gene expression pattern. The length of bars represents one standard deviation. The plots show results for 200 evolving populations. (2.95 MB TIF) [file pcbi.1000719.s001.tif]

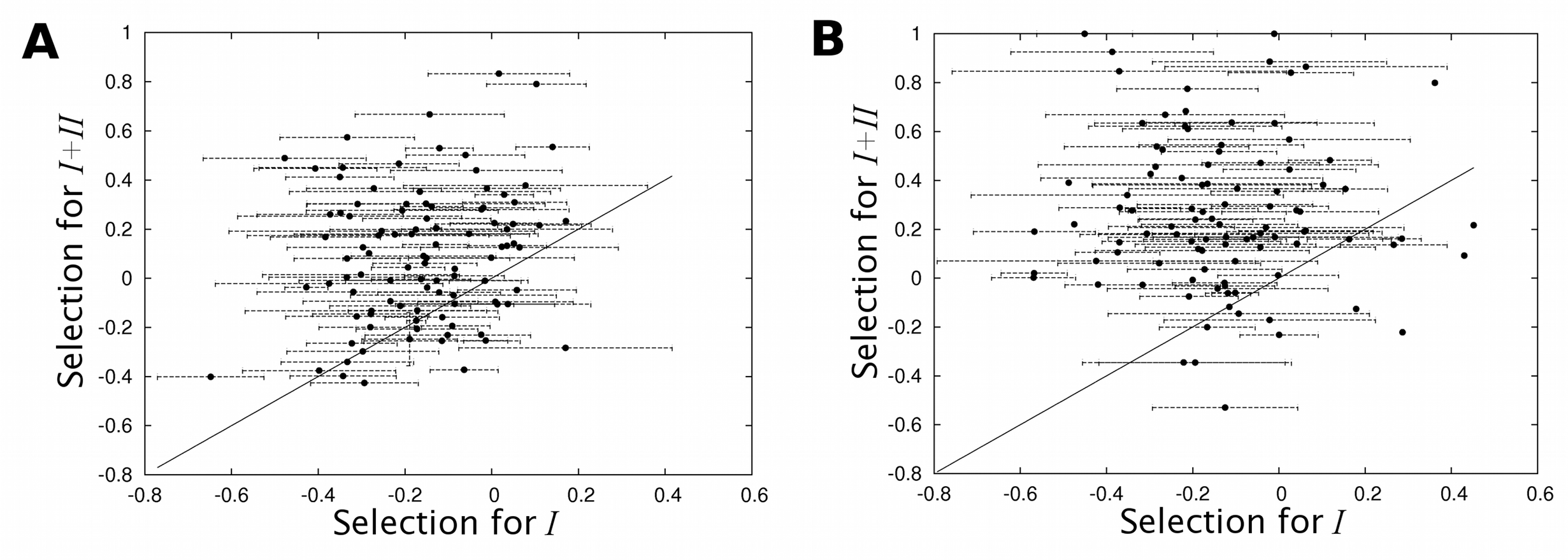

Supplement: Figure S3 — Modularity increases under evolution with different mutation rates. The horizontal axes indicate modularity in the best adapted networks after 1000 generations of selection for gene activity pattern I. The length of bars represents one standard deviation. The plots show results for 100 evolving populations. (A) Modularity increases using a mutation rate that equals half of the value used in other simulations (μ = 0.025; Wilcoxon signed-rank test; z = 6.8835; p = 2.9194×10^−12^). This increase occurs but requires longer time scales to achieve adaptation. The vertical axis shows modularity in the best adapted networks after an additional 3000 generations of selection for both gene activity patterns I and II. (B) Modularity increases using a mutation rate that doubles the value used in other simulations (μ = 0.1; Wilcoxon signed-rank test; z = 7.4921; p = 3.3862×10^−14^). The vertical axis shows modularity in the best adapted networks after an additional 1500 generations of selection for both gene activity patterns I and II. (1.48 MB TIF) [file pcbi.1000719.s003.tif]

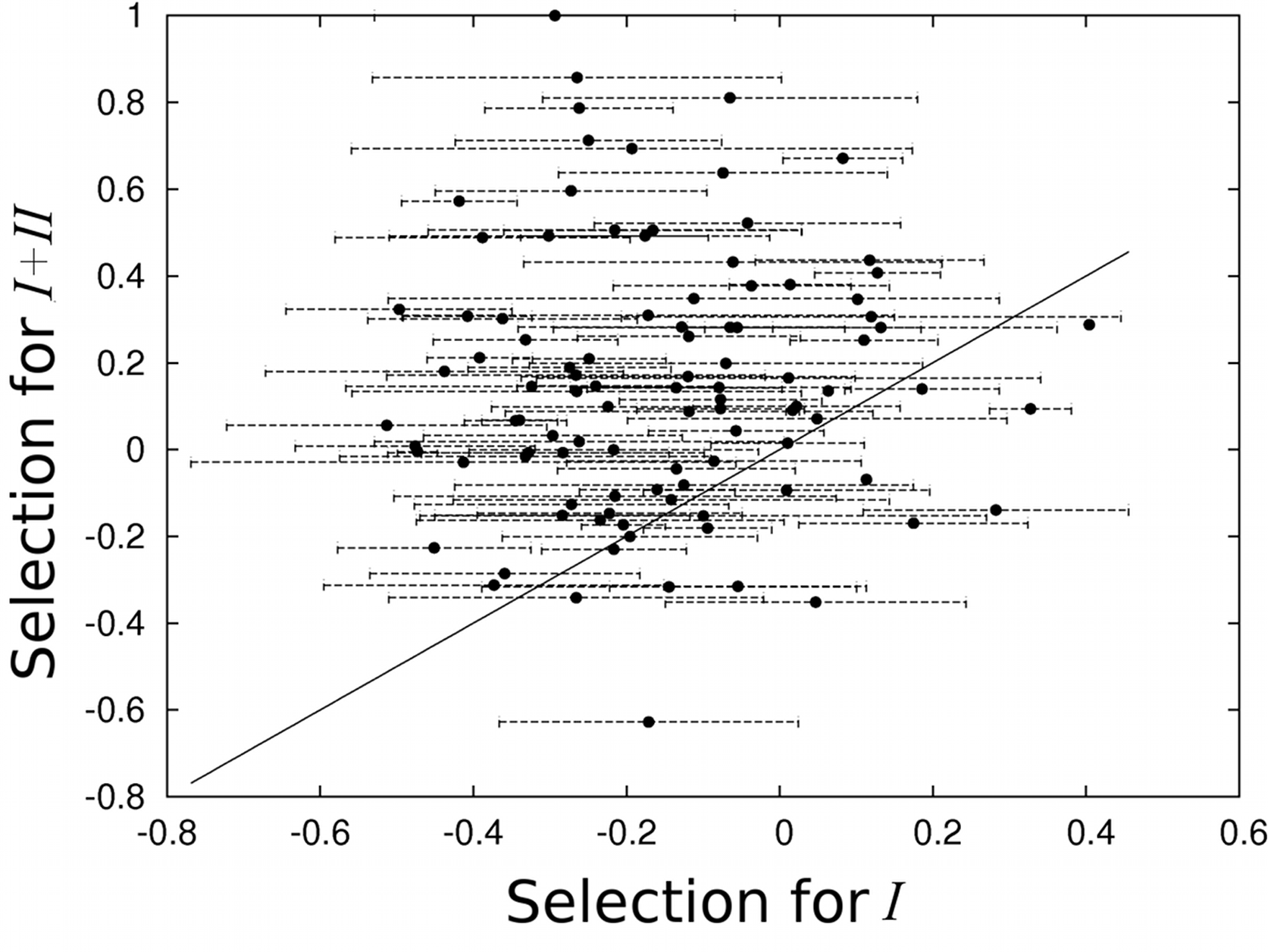

Supplement: Figure S4 — Modularity increases when fitness components related to each activity pattern combine multiplicatively instead of additively. Wilcoxon signed-rank test; z = 6.9385; p = 1.9809×10^−12^. The horizontal axis indicates modularity in the best adapted networks after 500 generations of selection for gene activity pattern I. The vertical axis shows modularity in the best adapted networks after an additional 1500 generations of selection for both gene activity patterns I and II. The length of bars represents one standard deviation. The plot shows results for 100 evolving populations. (0.83 MB TIF) [file pcbi.1000719.s004.tif]

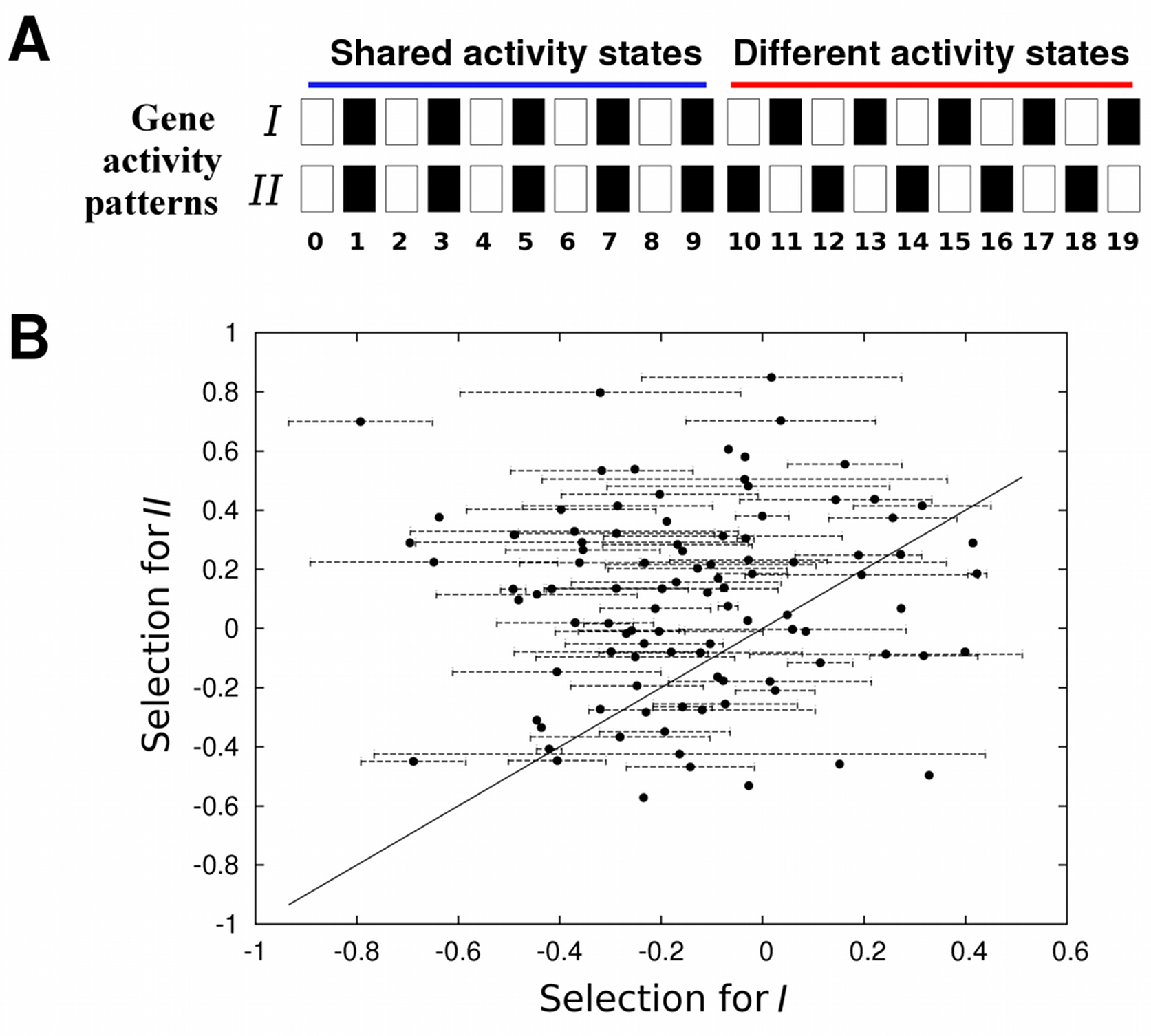

Supplement: Figure S5 — Modularity increases when evolving networks composed of twice as many genes as in other simulations. N = 20; Wilcoxon signed-rank test; z = 5.1987; p = 1.0032×10^−7^. (A) Activity patterns I and II share the activity state of genes 0–9, but show different activity patterns for genes 10–19. White squares represent active genes and black squares represent inactive genes. (B) The horizontal axis indicates modularity in the best adapted networks after 800 generations of selection for gene activity pattern I. The vertical axis shows modularity in the best adapted networks after an additional 2700 generations of selection for activity patterns I and II. We adjusted the mutation rate μ so that the expected number of individuals without any mutation is approximately the same as in all other simulations. Because of computational cost, we here followed 250 developmental trajectories for each network to evaluate the contribution to fitness associated to a certain gene activity pattern, instead of 500 as in our other analyses. The length of bars represents one standard deviation. The plot shows results for 100 evolving populations. (1.70 MB TIF) [file pcbi.1000719.s005.tif]

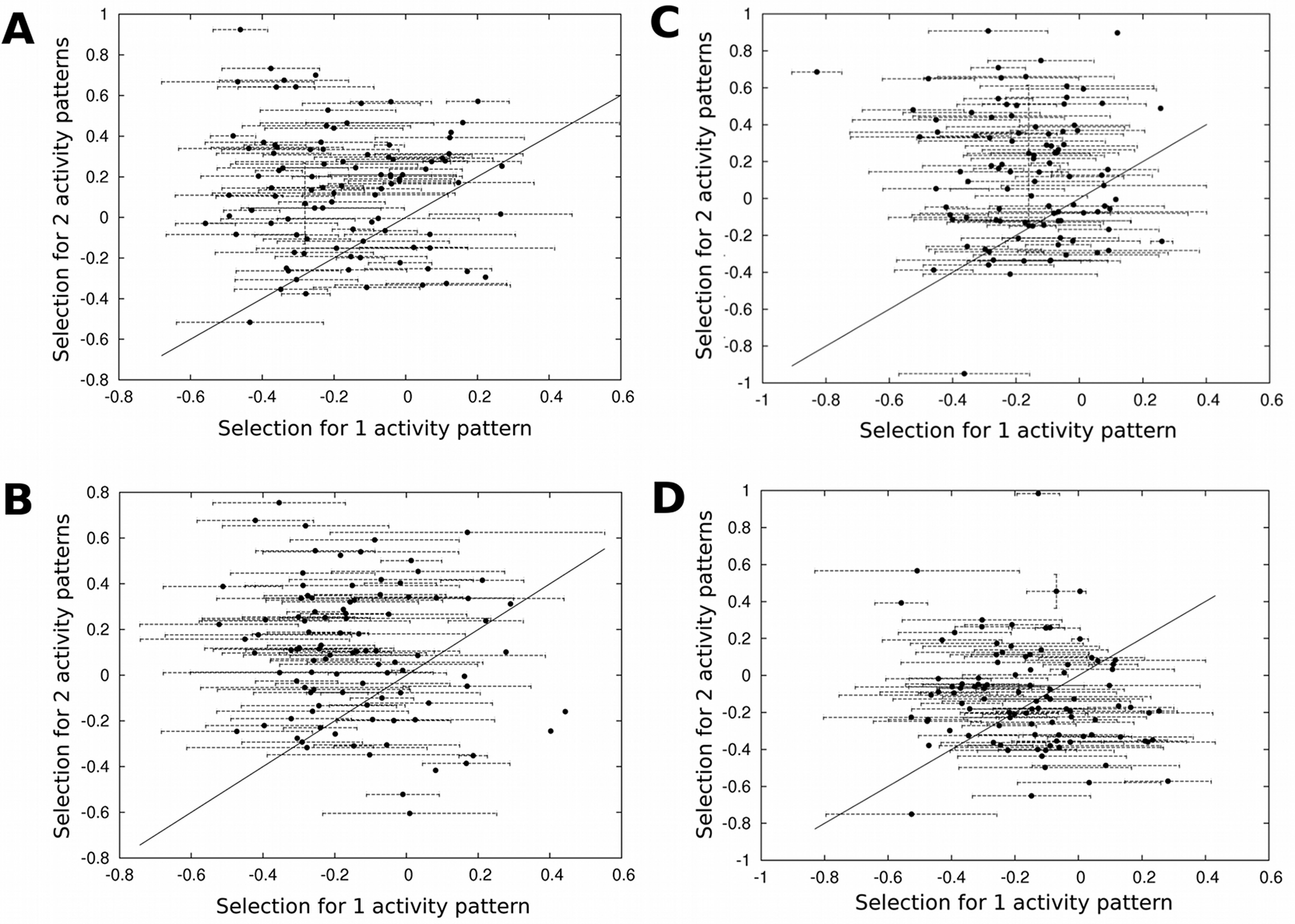

Supplement: Figure S6 — The increase in modularity does not depend on the identity of the selected activity patterns. The horizontal axes indicate modularity after 500 generations of selection for a single gene activity pattern. The vertical axes show modularity after an additional 1500 generations of selection for two gene activity patterns. The length of bars represents one standard deviation. The plots show results for 100 evolving populations. (A) Modularity increases in the best adapted networks when the two selected gene activity patterns differ in the activity state of 3 genes (Wilcoxon signed-rank test; z = 6.7185; p = 9.1811×10^−12^). (B) The same occurs when the two selected gene activity patterns differ in the activity state of 7 genes (Wilcoxon signed-rank test; z = 5.6045; p = 1.0445×10^−8^). (C) Modularity increases after selection for two gene activity patterns picked at random (Wilcoxon signed-rank test; z = 5.9449; p = 1.3834×10^−9^). We discarded pairs of gene activity patterns with less than two different activity states. The probability of picking a pair with k activity differences in a 10-gene network is p(k) = C10k0.510, where CNk is the binomial coefficient. However, after discarding activity patterns with less than two different activity states, p(k) = [C10k0.510][1−(C100+C101)0.510]−1. (D) Modularity does not increase when gene activity patterns differ in the activity state of all genes (Wilcoxon signed-rank test; z = 1.0281; p = 0.15196). This result is not due to a lack of adaptation, since networks that can attain both activity patterns in a stable manner arise in all evolving populations. (3.14 MB TIF) [file pcbi.1000719.s006.tif]

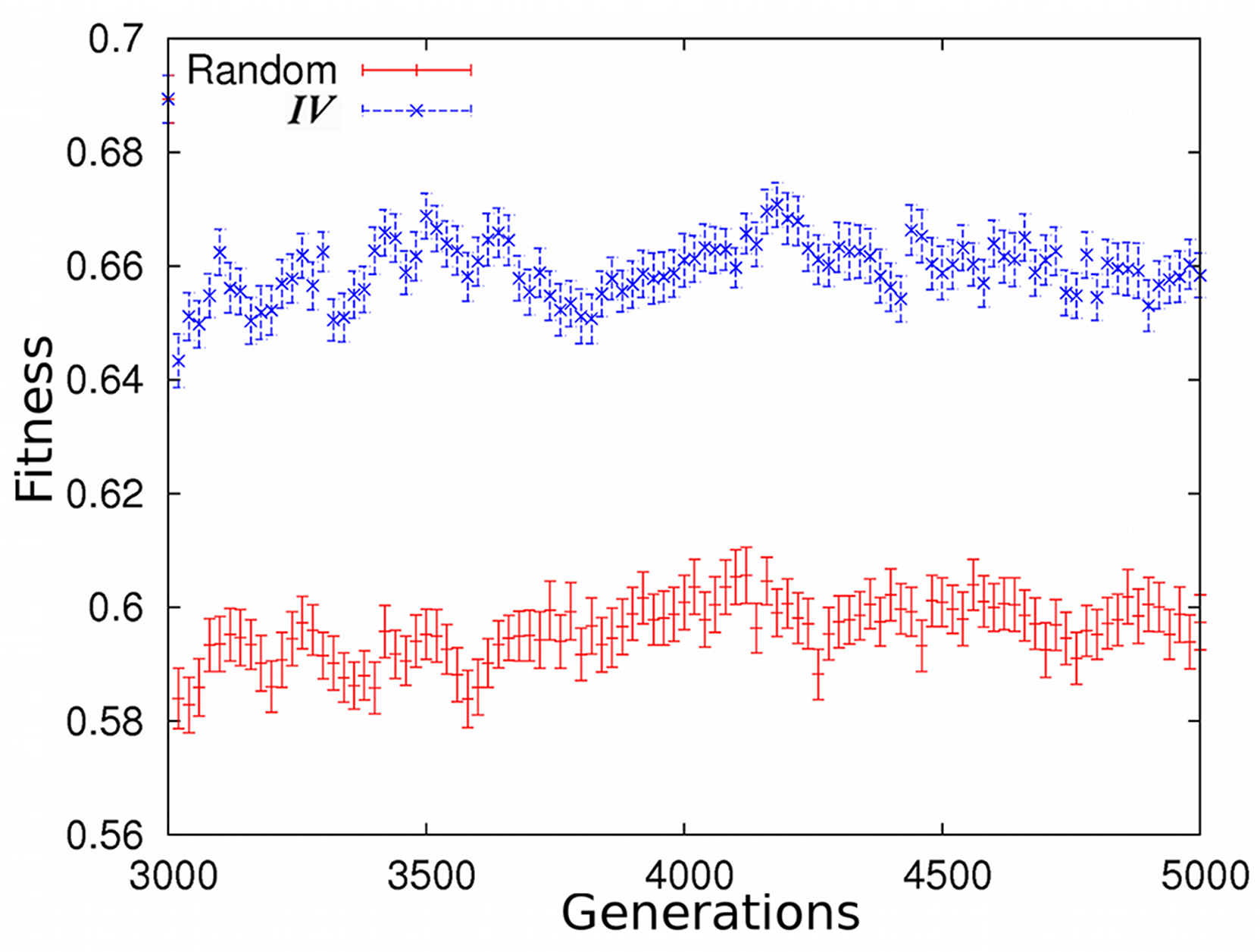

Supplement: Figure S7 — Mean fitness increases faster when co-option of existing gene activity states is possible. Mean population fitness increases faster when selecting for a new gene activity pattern (IV in Figure 5 in the main text) that co-opts activity states matching those of previously evolved modules than when such activity pattern is picked at random. This shows that the increase in fitness when selecting for pattern IV permeates the whole population, and affects not only the best adapted networks. The length of bars represents one standard error. (1.02 MB TIF) [file pcbi.1000719.s007.tif]

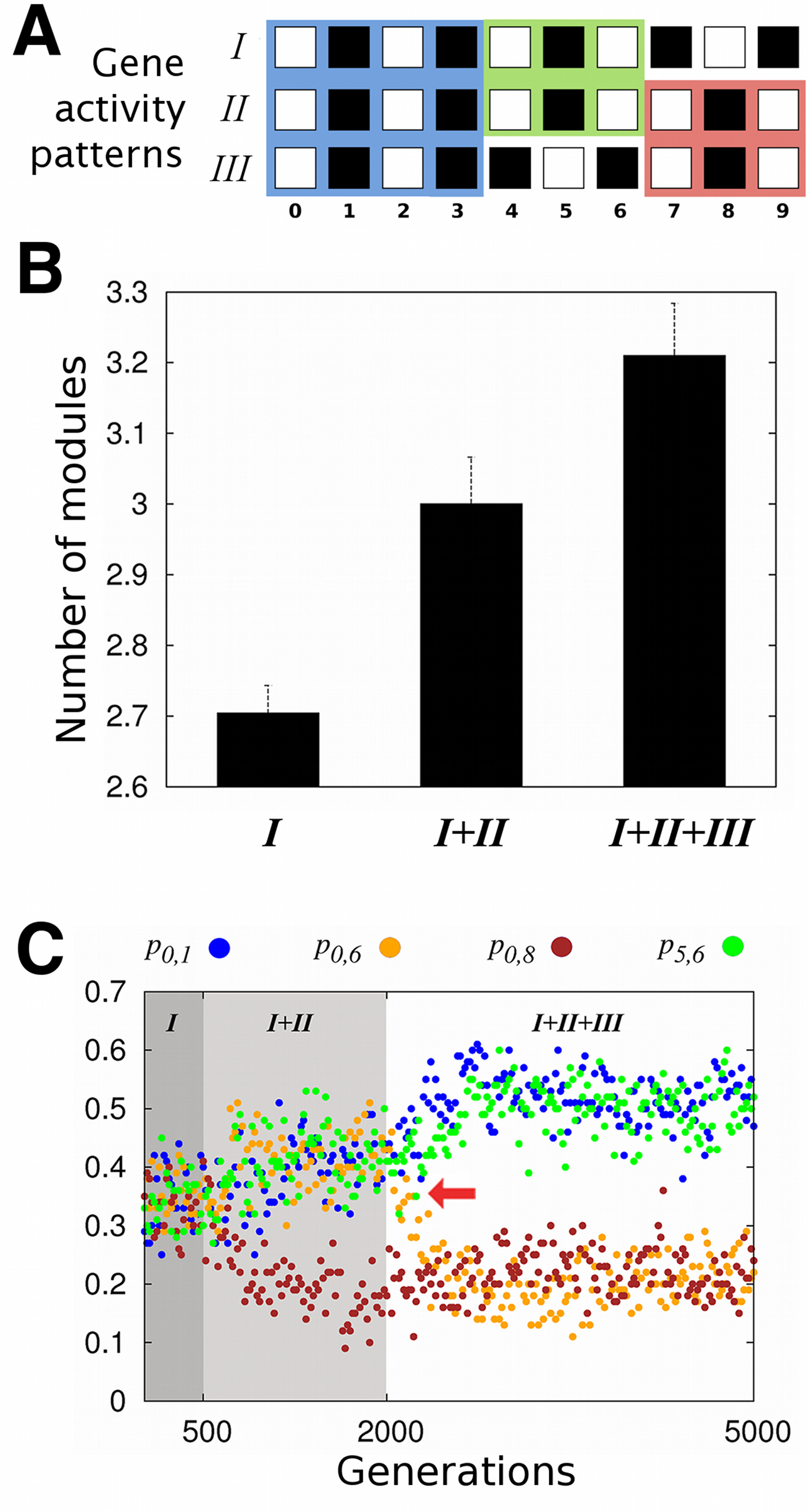

Supplement: Figure S8 — New modules arise after selection for a third additional pattern. (A) Gene activity patterns I, II and III, as in Figure 4A. (B) The number of modules in the networks with the highest fitness in each population, averaged across populations, increases after selection for the new additional patterns. The length of bars represents one standard error. (C) px,y stands for the frequency with which genes x and y occur in the same module in the networks with the highest fitness of each evolving population. When selection for a new activity pattern causes the activity of two genes to cease changing concertedly across the selected patterns, the probability of such genes lying in the same module decreases rapidly. This is the case of genes 0 and 8 after selection for activity pattern II starts, and also of genes 0 and 6 after selection for pattern III begins (red arrow). The plots show results for 100 evolving populations. (4.06 MB TIF) [file pcbi.1000719.s008.tif]
